# Isolated Langerhans Cell Histiocytosis of the Vulva in a 28-Year-Old Lady: A Report of a Case and Brief Review of the Literature

**DOI:** 10.1155/2022/8483008

**Published:** 2022-06-28

**Authors:** Maryam Sadat Sadati, Nafiseh Todarbary, Fatemeh Sari Aslani, Maryam Hadibarhaghtalab

**Affiliations:** ^1^Molecular Dermatology Research Center, Shiraz University of Medical Sciences, Shiraz, Iran; ^2^Dermatology Department, Shiraz University of Medical Sciences, Shiraz, Iran; ^3^Student Research Committee, Shiraz University of Medical Sciences, Shiraz, Iran; ^4^Pathology Department, Shiraz University of Medical Sciences, Shiraz, Iran

## Abstract

Langerhans cell histiocytosis (LCH) is a rare, proliferative disorder of Langerhans' cells. The presentation can vary from single organ involvement to multisystem and disseminated in severe cases, affecting children more than adults. Isolated vulvar involvement of LCH in a 28-year-old woman has rarely been described and also there are limited data for diagnosis and treatment. Herein, we report the case of a 28-year-old woman with isolated vulvar LCH, misdiagnosed with herpes simplex infection, successfully treated with thalidomide.

## 1. Introduction

According to the latest version of the National Comprehensive Cancer Network (NCCN), LCH is categorized to unifocal and multisystem (multifocal single-system or unifocal LCH involving critical organs). Unifocal LCH consists of isolated bone involvement, isolated skin disease, and involvement of other systems, except for critical organs. Multisystem LCH includes asymptomatic with no impending organ dysfunction, symptomatic or impending organ dysfunction, and pulmonary LCH [1].

In medical pieces of literature, only a few cases have been previously reported with vulvar LCH [2]. Primary isolated LCH of the female genital tract is rare, and less than 40 cases have already been reported worldwide [3]. This report presents the clinical and histopathologic features, diagnostic workup, and treatment of a 28-year-old woman as a vulvar LCH case from the Dermatology Department of Faghihi Hospital in Shiraz, Iran.

## 2. Case Presentation

**Figure 1:**
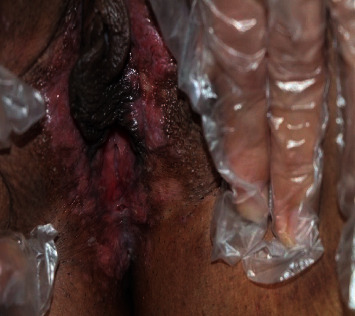
Pinkish mild erosions and small punched-out ulcers in genital area.

The patient was a 28-year-old lady admitted to the Dermatology Department of Faghihi Hospital with a chief complaint of small punched-out ulcers located in the genitalia area concomitant with pruritus and posturination dysuria since two years before. Her physical examination was normal except for mild pinkish erosions and two punched-out ulcers in the genital area (Figure 1).

Firstly, the clinical diagnosis was genital herpes simplex and the patient had received several courses of acyclovir and cryotherapy, but she was not cured. Lesion biopsy and immunohistochemistry were done after no improvement in the clinical practice.

**Figure 2:**
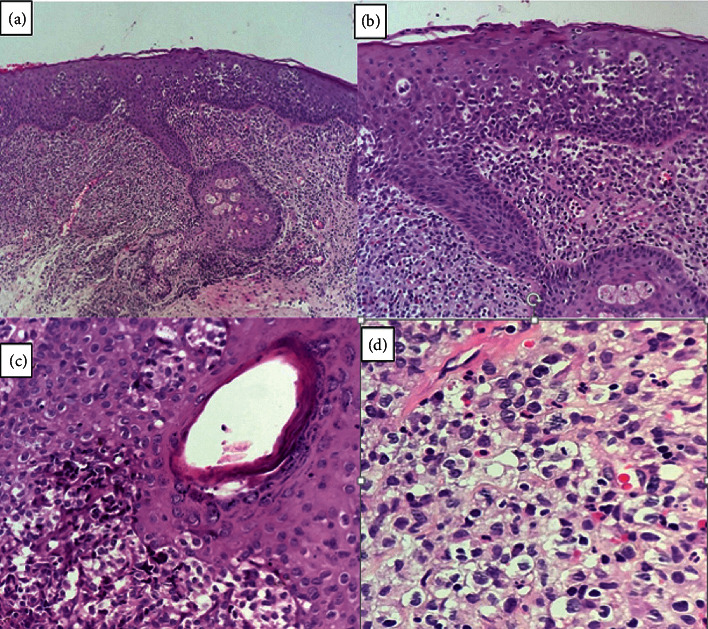
(a). There are some aggregates of Langerhans cells in the epidermal layer and diffuse upper and mid-dermal predominantly Langerhans cell infiltration (H&E × 100) (b). There are some aggregates of Langerhans cells in the epidermal layer and diffuse dermal predominantly Langerhans cell infiltration admixed with some eosinophils and few lymphocytes (H&E × 200) (c). Follicular epithelium involved by Langerhans cells (H&E × 400) (d). There is diffuse upper dermal predominantly Langerhans cell infiltration with mitotic figures admixed with some eosinophils and neutrophils and a few lymphocytes (H&E × 400).

Pathology results showed that the collected biopsy was 0.3 × 0.3 × 0.3 cm creamy-gray color skin favoring erosive lichen planus (LP) and lichen simplex atrophicus (LSA). Pathological findings were parakeratosis with some neutrophils and moderate acanthosis with multifocal aggregates of Langerhans cells. The upper and mid-dermis showed sheets of uniform ovoid cells with eosinophilic cytoplasm with a coffee bean or reniform nuclei and eosinophil and lymphocyte infiltration. The follicular epithelium was involved by some aggregates of Langerhans cells (Figure 2).

Based on oncology consultation, brain MRI, PET scan, bone marrow biopsy, skull, and chest X-ray were performed, all of which were normal.

**Figure 3:**
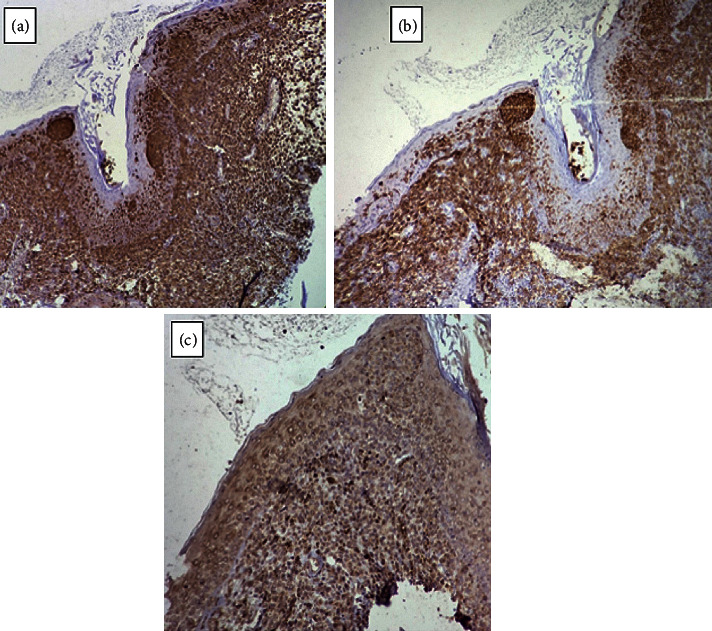
(a). S100 immunostaining shows strong diffuse positivity in upper dermis and scattered single as well as aggregates of Langerhans cells in the *epidermis* (×100) (b). CD1a immunostaining of Langerhans cells shows strong diffuse positivity in the upper dermis and scattered single as well as aggregates in the *epidermis* (×100) (c). CD68 immunostaining shows scattered weak positivity upper dermis (×100).

Immunohistochemistry profile on formalin-fixed and paraffin-embedded lesion of genitalia biopsy was CD1a positive, CD68 positive, and S100 positive, and the Langerin marker (CD207) was not available. Finally, the probable diagnosis was LCH (Figure 3).

**Figure 4:**
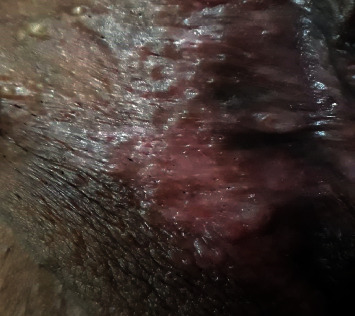
Remission after treatment by thalidomide.

Treatment was started with the administration of nitrogen mustard and fluocinolone, but she was not cured. Then, thalidomide, 100 milligrams once a day, was prescribed. The patient did not have a pregnancy plan by taking OCP pills and using condoms. Adverse effects and prohibition of pregnancy were explained to her in detail. Finally, the patient's lesions disappeared after one year (Figure 4). The patient was visited every three months for physical examination and checking lab data such as complete blood tests, pregnancy tests, and kidney and liver function tests until the end of treatment; then, she was visited annually.

## 3. Discussion

LCH is a rare disease, especially among adults, and it can occur at any age [4]. The most common sites of involvement consist of skin, bones, lymph nodes, bone marrow, hypothalamic-pituitary axis, lungs, spleen, liver, bowel, and orbit. In addition, LCH can involve different sites of the female genital tract, including the vulva, vagina, cervix, uterus, and ovaries [5].

In the case of LCH, workups such as full blood count and chemistry, thyroid function tests, urine analysis, and coagulation studies are usually requested. Additionally, radiographic examinations such as skull series and chest radiography should be done depending on the basic diagnostic tests and the 'patient's symptoms [6].

In 1939, Lane and Smith reported the first case of lower female genital tract LCH in a six-year-old child [5]. Herein, we reported a case of adult vulvar LCH with a mimic of herpes simplex lesions which can easily be misdiagnosed; therefore, having this clinical presentation with no response to routine treatment and no risk factor of LCH cannot put LCH off your mind.

Several neoplastic diseases can be mistaken for LCH, such as malignant melanoma, squamous cell carcinoma, sarcoma, and 'Paget's disease of the vulva. Systemic diseases in any patient suspected of vulvar LCH should be evaluated [5].

Although this condition is rare, taking a detailed and accurate medical history, accurate physical examination, and necessary workups like tissue biopsy are strongly recommended to prevent misdiagnosis of the disease and delay in beginning the proper treatment.

Vulvar involvement of LCH can be treated by topical and oral steroids, chemotherapy (vinblastine, vincristine, or 2-chlorodeoxyadenosine), immune modulators (methotrexate or tacrolimus), radiation, partial or radical vulvectomy with or without lymph node resection, thalidomide, and radiation therapy [7].

Considerably, treatment of isolated vulvar LCH necessitates more investigation, and there is a lack of evidence for standard therapy in patients with pure genital involvement. Santillan et al. reported the first successful administration of thalidomide for genital LCH treatment [5]; it is a successful treatment for localized LCH such as genital, perianal, and disseminated skin lesions [8–10].

The exact mechanism of thalidomide in treating isolated vulvar LCH is not completely recognized. However, recent studies demonstrated anti-inflammatory and antineoplastic features by inhibiting the production of IL-6 and TNF-alpha. These cytokines had expressed extensively within the biopsy of LCH lesions; therefore, they may play a vital role in the tumor cells' relapse, viability, and irregular maturation. In addition, the beneficial effect of thalidomide is possibly due to balancing the expression of pathological cytokines in LCH [11].

Thalidomide is a safe and well-tolerated drug for isolated genital LCH that can cure mucocutaneous LCH and prevent relapse. In our case, our patient's administration of thalidomide, 100 milligrams daily, was effective, easy to administer, and well-tolerated, and lesions were cured.

However, thalidomide is beneficial in vulvar LCH. Due to teratogenic side effects, the patient must have close follow-ups [9]. It could also have a poor response in high-risk multisystem diseases. Generally, thalidomide, 100 mg per day, is used in adults, but monitoring for toxicity with peripheral neuropathy is necessary [12].

## Data Availability

Data are available on request by contacting the corresponding author: maryam_hadibarhaghtalab@yahoo.com.
